# Pressure Injuries: prevention, treatment, and complications – Part II^؆^

**DOI:** 10.1016/j.abd.2025.501215

**Published:** 2025-10-27

**Authors:** Bruna Cristina Velozo, Michelle Venâncio Hong, Larissa Cassiano Bernardo, Meire Cristina Novelli e Castro, Jose Contreras-Ruiz, Luciana Patricia Fernandes Abbade

**Affiliations:** a Nursing Department, Faculty of Medicine, Universidade Estadual Paulista, Botucatu, SP, Brazil; b Centro Dermatológico Polanco Los Cabos, Los Cabos, Mexico; c Hospital General Dr. Manuel Gea González, Ciudad de México, Mexico; d Department of Infectology, Dermatology, Imaging Diagnosis and Radiotherapy, Faculty of Medicine, Universidade Estadual Paulista, Botucatu, SP, Brazil

**Keywords:** Dressings, Pressure ulcer, Wound Healing

## Abstract

Pressure injuries must be identified and managed early to prevent them from becoming difficult to heal. In this review, the main complications inherent to pressure injuries will be highlighted, as well as the expected prognosis for patients with this health condition. Furthermore, the most current preventive measures are emphasized and supported by robust references from systematic reviews and guidelines. In the treatment section, an updated and comprehensive conceptual model is presented to guide dermatologists and healthcare professionals in assessing injuries in general, as well as pressure injuries. The focus on the debridement and management of infections and biofilm is emphasized. The importance of preparing the wound bed and the peri-wound area will also be addressed, unifying concepts with the best scientific evidence.

## Introduction

Pressure injuries (PIs), also known as “pressure ulcer”, are considered chronic and should be identified and managed early to prevent them from becoming difficult to heal.^1^ Their high incidence and global prevalence,^2^ make prevention a current concern and necessity on the agendas of healthcare organizations.^3^ PIs have a significant economic impact related to both direct and indirect costs involved in their management, such as costs for wound care materials, nursing time, surgery, debridement, additional hospitalization time, medications, laboratory and imaging tests, and secondary prevention measures (to prevent further deterioration, occurrence, or recurrence of PIs), treatment of complications, and medical consultations.^4^ In addition, there are unquantifiable expenses related to the negative impact of PIs on patients and their families.^5^

Dermatologists may contribute significantly to the care of patients with PIs because their training makes them valuable members of the wound care team through the appropriate diagnosis of PIs, indicating diagnostic tests necessary for evaluation, recommending correct care for the surrounding skin, and indicating proper treatments and specialized dressings. They may also guide strategies for the prevention and treatment of complications. Thereby saving resources for the healthcare system and improving the quality of life of the patients.^6^

In Part I, PIs general concepts, clinical aspects, and laboratory findings were updated. Part II focuses on its prevention, treatment, and complications.

## Prevention of pressure injuries

### Risk assessment for pressure injuries

The risk assessment for developing PIs should be structured, including a valid and reliable risk prediction scale, a thorough skin assessment, and a critical interpretation of the results.^7^ The latter has been emphasized as a key element in identifying potential risk factors that the scales may not cover.^8^

Risk assessment for PIs assists in the selection of effective preventive interventions, including the frequency of skin assessment and repositioning. Some interventions are directly related to specific risk factors (e.g., managing moisture when present), while others are assigned based on the overall estimated risk score.^9^ Thus, risk prediction scales highlight vulnerable areas, reinforce the importance of continuous assessment, and support preventive mechanisms.^10^ In addition, the scales can contribute to epidemiological studies by identifying groups of individuals at risk. Ideally, risk prediction scales for PIs should be used specifically for the population for which they were developed.^7^ For adult patients in general, the gold standard is currently the Braden Scale,^11^ which was translated into Brazilian Portuguese in 1999,^12^ it covers six risk domains: mobility (ability to change and control body position); activity (degree of physical activity); sensory perception (ability to respond appropriately to discomfort related to pressure); moisture (degree of skin exposure to moisture); friction and shear (horizontal forces acting in conjunction with the vertical pressure); and nutrition (usual dietary intake pattern). These domains are scored from one to four (except the last one, which is scored from one to three). The score ranges from six to 23, classifying individuals based on the total score obtained as follows: no risk (score 19‒23), low risk (score 15‒18), moderate risk (score 13‒14), high risk (score 10‒12), and very high risk (score ≤ 9).^12^

The Braden-Q Scale was modified for children, specifically developed for neonates up to 18-years-old.^13^, ^14^ More recently, for neonates, the Braden QD Scale has been introduced,^15^ and it has already been adapted for Brazil.^16^ The Glamorgan Scale,^17^ developed for hospitalized children and adapted to Portuguese, has demonstrated higher accuracy than the Braden-Q.^18^ More recently, it has been applied to pediatric Intensive Care Units (ICUs).^19^

Patients are at high risk for the development of PIs during surgical procedures. The first scale developed to assess this risk was the Munro Scale.^20^, ^21^ However, in addition to the intrinsic characteristics of the patient, surgical positioning during the perioperative period becomes the most important challenge in preventing PIs. The ELPO Scale^22^ developed by a Brazilian nurse for this purpose, is widely used and validated, and utilized in other countries.^23^, ^24^ Recently, a new scale called PRASI (Proactive Risk Assessment for Skin Integrity) has been introduced, primarily assessing skin integrity.^25^

For critically ill patients in ICUs, the most studied scales currently, which have already been adapted to Portuguese, are: EVARUCI,^26^, ^27^ CALCULATE^28^, ^29^ and Cubbin & Jackson.^30^, ^31^ Studies have shown that these scales have superior risk prediction compared to the Braden Scale.^32^, ^33^ In agreement, a systematic review from 2024,^34^ highlighted that the use of the Braden Scale in ICUs is not recommended due to the inconsistency of its psychometric properties, as it does not fully address the risk factors for this patient profile.^34^

### Preventive measures

In addition to scales and clinical judgment, prevention bundles have become more widespread. These are a set of evidence-based interventions that, when implemented together, aim to impact patient outcomes,^35^, ^36^ including risk assessment, moisture control, patient mobility, nutrition, support surfaces,^7^ and team training.^37^ However, in a systematic review, studies on bundles showed bias, particularly in the post-intervention assessment of the application of these measures, indicating that further research is needed to evaluate their effectiveness.^38^

Clinical awareness and appropriate resource allocation are necessary to prevent PIs.^39^ Minimizing compressive (pressure) and shear forces at the interface between the body and the support surface or between the body and a medical device are valid clinical interventions to reduce the risk of developing PIs.^7^

**Fig. 1:**
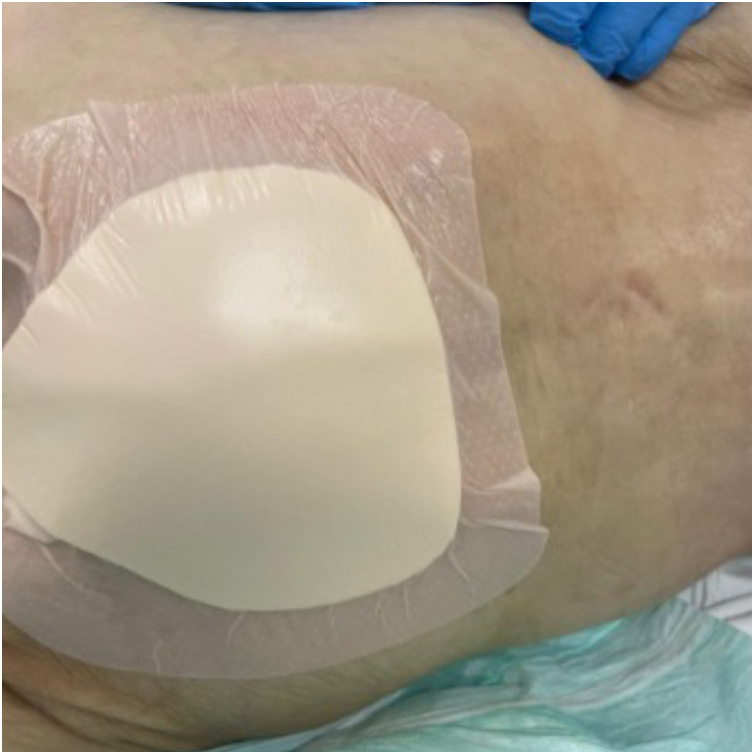
Prophylactic polyurethane foam and silicone dressing on the sacral region in a patient at high risk for developing pressure injuries.

Table 1 lists the main preventive measures recommended for patients at risk of developing PIs, including evidence related to skin assessment and care,^7^ and preventive dressings (Fig. 1), repositioning,^7^, ^37^, ^44^, ^45^ bed head,^7^, ^40^, ^46^ surfaces (bed and mattresses),^7^, ^40^, ^47^, ^52^ and nutrition.^7^, ^40^, ^48^, ^49^, ^50^, ^51^, ^53^

**Table 1:** Main preventive measures for patients at risk of developing pressure injuries (PIs) and their respective recommendations.^7^, ^36^, ^37^, ^40^, ^41^, ^42^, ^43^, ^44^, ^45^, ^46^, ^47^, ^48^, ^49^, ^50^, ^51^, ^52^, ^53^

Preventive Measures	Recommendations
Skin Assessment and Care	• Daily skin assessment in areas of bony prominences and medical devices, paying attention to areas of erythema, oedema, or changes in skin temperature or texture.
	• Keep the skin clean and hydrated, avoiding the use of alkaline soaps.
	• Avoid rubbing and vigorously massaging skin at risk of pressure injuries, especially over bony prominences.^7^
	• Use a barrier cream or barrier spray, especially in areas with persistent moisture exposure, such as the diaper area in incontinent patients.
	
Preventive Dressings^a^	• Recommended for all patients at risk of PIs, especially those in intensive care units, in areas of bony prominences.
	• Use silicone and multilayer foam dressings, especially on the sacral area and heels.
	
Repositioning	• The exact interval for repositioning is not known and is done empirically.
	• When repositioning, consider individual factors such as skin tolerance, overall medical condition, treatment goals, comfort, and pain.
	• Repositioning every two hours has been reported in some studies to contribute to a reduction of up to 25% in the incidence of PIs^37^, ^44^ having a specific team (turning team) to perform repositioning has been shown to reduce this risk by 51%.^44^
	• On the heels, a foam pillow or cushion should be used to distribute the weight of the leg along the calf without applying pressure to the Achilles tendon and the popliteal vein.
	• Manual techniques and equipment, such as rotation mattresses and sliding sheets, assist in proper repositioning, reducing friction during movement, and helping with comfort.
	• When repositioning, consider individual factors such as skin tolerance, overall medical condition, treatment goals, comfort, and pain.
	
Head of bed^b^	• Ideally, the head of the bed should be kept at the lowest elevation possible, consistent with clinical conditions and other constraints.
	• When elevation of the head of the bed is necessary, precautions should be taken to avoid patient sliding or migration.
	
Surfaces (bed and mattress)	• When choosing the best surface, individual patient characteristics should be considered, including the risk of PIs.^40^
	• Choose mattresses that allow a minimum thickness of 2.5 cm between the patient’s body and the rigid surface of the bed.
	• Evaluate mattress density to ensure the patient does not “sink” into the mattress.
	• Recommended mattresses: pneumatic mattresses (air-alternating mattresses) offer better cost-effectiveness compared to foam mattresses (pyramid-shaped or “egg crate” type).^52^
	
Nutrition	• Nutritional status is a significant factor in preventing PIs, as both malnourished lean and obese patients face a heightened risk of developing them due to compromised tissue integrity and reduced healing capacity.^53^
	• Screen all patients for nutritional risk using a validated tool, such as the Malnutrition Universal Screening Tool (MUST).^49^
	• Nutritionist assessment to develop an appropriate plan for at-risk patients.
	• For neonates and children with or at risk of PIs who have inadequate oral intake, consider enriched foods, age-appropriate nutritional supplements, or enteral or parenteral nutritional support.
	• Supplements, in general, still have low evidence in prevention and require studies on special diets (e.g., high-protein, vegetarian).^51^

a According to a systematic review,^37^ foam dressings have been shown to reduce the incidence of pressure injuries by 30%.

b Elevating the head of the bed creates higher interface pressures between the skin and the bed surface at the sacrococcygeal area. Mechanically ventilated patients, when at a 45 ° elevation, have almost a 50% higher risk of pressure injuries compared to a 30 ° elevation.^46^

## Treatment of pressure injuries

The first step in the care pathway for PIs is to relieve pressure in affected areas and prevent the development of injuries in new areas. The treatment of a PI should occur at the earliest stage to prevent the development of hard-to-heal or chronic wounds, which do not reduce by 40%‒50% in size within four weeks^54^ when treated with a good standard of care.^55^ Moreover, the treatment of PIs should focus on the underlying cause, which has been explained earlier and is directly related to the factors that led to the injury, the most important of which is usually pressure. All pressure on the injury should ideally be removed. In many cases, this may be impossible, and therefore, optimization of the patient´s environment is necessary. An essential intervention is pressure relief and load redistribution through frequent repositioning.^7^ Thus, physical measures are necessary to remove mechanical loads and redistribute pressure in order to prevent additional risks and tissue damage, and to promote healing.^56^ Some patients, due to severe conditions, may have compromised repositioning. Therefore, the dressing of a PI, in addition to promoting healing, should also interfere with the loading state in the soft tissues at and around the wound. An adequate dressing with a protective biomechanical effect should absorb compressive and shear deformation energy from the surrounding tissues and the wound itself, alleviating the transferred loads.^56^ Examples of biomechanical performance dressings are multilayered anisotropic ones, which aim for different resistances to compression or tension depending on the direction in which the force is applied. This type of dressing has proven effective in the prophylaxis of sacral PI^57^, ^58^, ^59^ and has also demonstrated efficiency with a focus on treatment, that is, when a PI already exists and is subjected to bodyweight forces.^56^

The improvement of patient mobility taught by physical therapists helps reduce incontinence and immobility, thereby decreasing the risk of PI development.^60^ An example is the treatment of PIs with electrical stimulation, where clinical trials have provided ample evidence of its effectiveness,^61^ as already recommended by international guidelines.^7^

Optimizing nutrition has not been shown to be definitive, but research has shown that specialized nutritional supplementation, in addition to standard wound care, can effectively improve the healing of PIs.^62^, ^63^ Therapeutic nutrition can support wound healing by increasing collagen production and helping to replenish the critical nutrients needed for healing.^64^ Patients with PIs are generally malnourished and protein-deficient, even those who are overweight or obese, as evidenced by low levels of nutritional biomarkers. Successful healing depends on collagen synthesis/deposition and epithelial resurfacing, both of which are highly reliant on adequate nutrient stores and support.^40^

Once the causes that originated or perpetuated the PIs have been addressed and corrected or improved, the next step is wound bed preparation or a local approach to the PIs.^65^, ^66^

When choosing the most appropriate local approach and dressing (primary and secondary), it is essential to consider the treatment objectives, which may include: protecting the wound bed during healing, promoting granulation tissue formation, supporting autolysis to aid in the debridement of devitalized tissue, managing local and systemic infection, controlling excess exudate and unpleasant odor, managing hypergranulation, and assessing the peri-wound.^67^ In line with this, it is essential to consider the size and depth of the wound, its classification and duration, and patient-related aspects such as emotional and social factors, access to healthcare, environment, and adherence to the treatment plan.^1^ Also, it is essential to consider the overall health and goals of the family and patients. Palliative care for PIs refers to the relief of symptoms and suffering related to wounds in the context of a serious illness. Moreover, it is essential to consider the overall health and goals of the family and patients.^40^ Injuries that occur at the end of life should focus on interventions for symptom management, patient comfort, and well-being, rather than on healing the wound.^68^ Topical pain treatments include viscous lidocaine gel and dressings that deliver ibuprofen and local opioids. Wound odor may be reduced with charcoal dressing, antiseptics, ionized silver dressings, or metronidazole gel. Alginates, gelling fibers, and absorbent foam dressings can manage excess drainage and protect surrounding skin from moisture-related damage.^69^

**Fig. 2:**
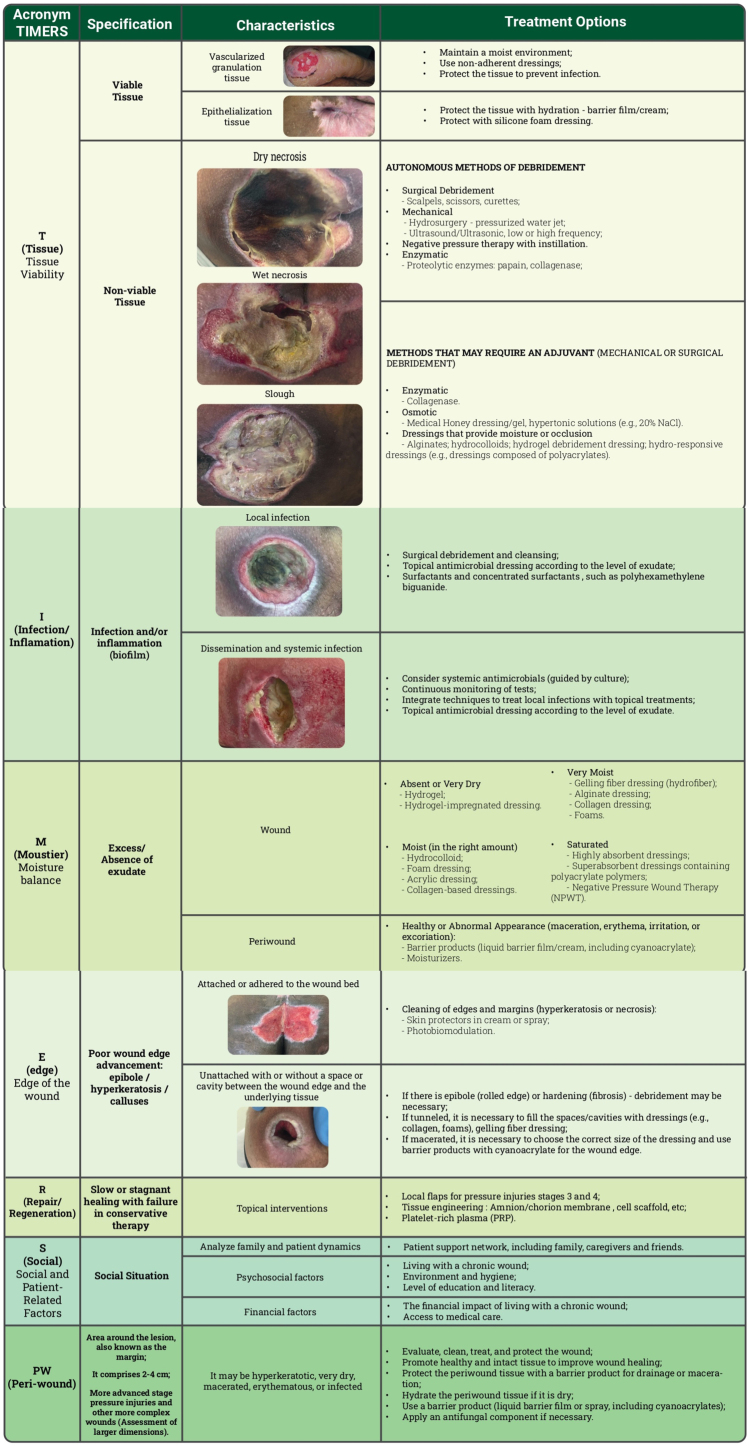
TIMERS adapted by adding the Peri-Wound approach (PW).^1^, ^55^, ^67^, ^70^

All these previously mentioned aspects have been incorporated into the acronym TIMERS (Fig. 2), essential for facilitating efficient wound care, focusing on rigorous assessment, and implementing interventions to optimize the healing process.^7^, ^55^

### T (Tissue): Assessment of Tissue viability in the injury bed

Assessment of tissue viability in the injury bed involves identifying devitalized or non-viable tissue, which hinders the healing process and facilitates infections.^1^ Removal of devitalized tissue (debridement) is essential, including slough, necrotic tissue, debris, toxins, microorganisms, and biofilm, both in the ulcer bed and in the peri-wound area, as well as hyperkeratosis.^70^

Cleaning or hygiene is the first step in the process. Its primary goal is to minimize microbial load and remove surface contaminants, debris, and microorganisms from the wound, aiming to establish a clean environment that reduces the risk of infection and promotes the formation of healthy granulation tissue.^65^, ^70^ Cleaning also ensures better visualization of the wound bed and access to non-viable tissue.^7^, ^70^ Proper hygiene should be performed on the wound bed and the peri-wound skin.^1^, ^55^ It is primarily recommended to use 0.9% sodium chloride solution or clean (drinking) water for irrigation.^71^

Therefore, in cases of hard-to-heal PIs, it is necessary to clean both the wound and the peri-wound area with antiseptic products suitable for skin (Ph 4–6), such as Polyhexamethylene Biguanide (PHMB).^71^

Debridement, on the other hand, is a critical step in wound bed preparation, aiming to remove barriers that impede the healing process.^65^ Its goal is to remove necrotic and sloughy tissue, reduce bacterial load, and eliminate biofilm.^1^ It contributes to removing fibroblasts and keratinocytes that undergo phenotypic changes (senescence), leading to chronic inflammation, fibrosis, and delayed healing.^72^ Thus, debridement converts chronic wounds stagnated in the inflammatory phase into an environment more similar to acute wounds, promoting healing.^1^, ^73^ The following non-viable tissues require management with debridement (Fig. 2):

• Necrotic tissue: Resulting from localized tissue death due to infection or ischemia. It may appear black, brown, or Gray, and can be dry (generally non-infected) or moist (often infected), and is usually adhered to the wound bed.
• Slough: A complex mixture of exudate proteins, degraded extracellular matrix proteins, white blood cells, and various microorganisms in planktonic and biofilm phenotypes.^70^

The different types of debridement are described in Fig. 2. Integrated debridement involves combining debridement methods according to the individual needs of the patient, preferences, and environment, as well as available resources and the skills of healthcare professionals.^70^ In addition, professionals need to be aware of the contraindications for each procedure. Surgical debridement, for example, should be carefully evaluated in patients on anticoagulants, those with a high surgical risk, or those who have ischemia, especially if the wounds are extensive.^1^ Other debridement methods, such as autolytic or mechanical debridement, are less invasive and require less training, making them more widely used.^70^ However, autolytic debridement (e.g., with hydrogels and hydrocolloids) is not the method of choice in cases of active infection with large amounts of devitalized tissue (e.g., gangrenous tissue). In these cases, surgical debridement should be considered.”

### I (Infection/Inflamation)

Infection and inflammation prevent healing and are crucial factors to be monitored.^74^

Local infection is characterized by the presence and proliferation of microorganisms within the wound, triggering a host response that often causes delayed healing.^75^ Local infection is confined to the wound and the immediate peri-wound area (less than 2 cm) and often presents with subtle signs and symptoms that may not be immediately recognized, such as hypertrophic granulation tissue, bleeding, friable granulation, epithelial bridges, formation of pockets in the granulation tissue, and increased exudation.^75^

Almost all chronic wounds contain biofilms, complex community structures surrounded by an extracellular polymeric substance secreted by various pathogenic bacteria to evade host defenses and increase resistance to antimicrobial drugs.^76^, ^77^ Combined with laboratory and clinical studies, biofilm in the wound can manifest as a thin layer of yellowish, gelatinous, and translucent material visible to the naked eye.^78^ Additionally, it can be present both on the surface and in deep tissues, stimulating chronic inflammation and delaying the healing process.^77^ Surgical debridement is the gold standard for removing biofilm, making the wound clean and conducive to repair by temporarily disrupting the mature biofilm.^77^ In addition, there is treatment with nanotechnology where dressings containing silver or zinc ions compete with the extracellular polymeric matrix of biofilms for binding sites, reducing the intercellular adhesion of pathogenic bacteria.^77^

In general, topical antibiotics alone are no longer recommended for wounds, as they may promote the development of bacterial resistance.^1^ Antiseptics rarely induce antimicrobial resistance.^1^ A wide range of antiseptics is available, like povidone iodine, cadexomer iodine, chlorhexidine, PHMB, silver (nanocrystalline or ionic), octenidine, reactive oxygen, and hypochlorous acid. However, according to a systematic review, the effects of topical antimicrobial treatments on PIs are not clear, and the quality of the evidence ranges from moderate to very low.^79^

Signs of deep infection, such as increased erythema, foul odor, warmth, increased exudate, fever, new or increasing pain, and elevated white blood cell count, should be treated with systemic antibiotics, and wound care should be done using topical treatments similar to those used for local infections.^75^ Other indicators, such as slow healing after two weeks of standard treatment, discoloration of granulation tissue to pale or dark tones instead of healthy red, and friable tissue, indicate possible infection in wounds.^40^

In disseminated infection, such as cellulitis, microorganisms invade the surrounding tissue, spreading from a lesion, and the signs and symptoms extend beyond the wound edges. Disseminated infection can involve deep tissues, muscles, fascia, organs, or body cavities, with possible signs of increasing induration, lymphangitis, crepitus, wound dehiscence with or without satellite lesions, and widespread inflammation or erythema greater than 2 cm from the wound edge.^75^ Systemic infection is the phase in which microorganisms spread throughout the body via the vascular or lymphatic systems, eliciting a host response. A local wound infection can also trigger the systemic inflammatory response through other pathways, such as toxin release or a dysregulated immune system. Symptoms may include malaise, lethargy or general nonspecific deterioration, fever/pyrexia, severe sepsis, septic shock, organ failure, and death.^75^

Routine wound culture is not recommended when there is no suspicion of infection. However, when a clinical diagnosis of disseminated and systemic infection is made, an ulcer culture method is recommended to confirm the initial diagnosis, identify resident microorganism species, and determine effective antibiotics to treat the wound. Current literature provides three laboratory techniques for diagnosing wound infections: deep tissue biopsy, needle aspiration, and swab culture. A blood culture should also be performed in the case of systemic infection.^75^

Deep wounds or those with bone exposure are susceptible to osteomyelitis, which is better detected by magnetic resonance imaging or bone biopsy, and requires intravenous antibiotic therapy,^1^ based on culture results.^40^ However, clinical diagnoses related to PIs have been discussed. A systematic review found that no alternative diagnostic method (clinical, microbiological, or radiological) is reliable for diagnosing osteomyelitis associated with pelvic PIs.^80^ Another systematic review^81^ describes osteomyelitis in the sacral region, which is the most affected area by PIs according to incidence studies.^2^ In this review, bone biopsy remains the only accurate diagnostic method, but osteomyelitis is relatively rare in patients with stage 4 sacral PIs.^81^ Furthermore, it indicates that short-term antibiotics can treat acute soft tissue infections, with a duration of 2- to 6-weeks depending on the infection's depth; there is no evidence to support extending treatment beyond 6-weeks.^81^ More recently, a review explains that evidence remains inconclusive for microbiological and histological diagnoses, suggesting that bone biopsy remains limited for patient management.^82^ This highlights the importance of understanding patient frailty and the underlying evidence for bone biopsy in order to consider changes in clinical management and the risk-benefit ratio.

### M (moisture): Assessment of the degree of exudation

One of the keys to wound care is maintaining the ideal moist environment, aiming to create a balanced setting that facilitates healing.^83^ However, when a wound exhibits either excessive or insufficient exudate production, it can hinder the healing process and cause damage to the surrounding skin.^1^

Considerations include the characteristics of the exudate regarding its quantity, color, odor, viscosity, potential constituents, and appearance.^84^ Purulent, hemorrhagic-purulent, or seropurulent exudate may indicate infection. Additionally, color and odor can indicate microbial influence in the wound, including biofilm and infection.^1^

Dressings are considered to have a unique potential for addressing issues related to exudate.^83^ Selecting a dressing that manages the exudate from PIs and protects the peri-wound skin, as maceration and continuous contact with exudate hinder the healing process.^40^ Viscous exudate requires different management than thin watery exudate, as products designed to handle thin exudate may become blocked by viscous exudate, reducing absorption and causing buildup in the dressing.^1^ Low-viscosity exudate can be managed using a variety of absorbent dressings, including foams, calcium alginates, gelling fibers, superabsorbent dressings, multilayer dressings, polymer pads, and Negative Pressure Wound Therapy.^1^

Low moisture levels can be treated with products that donate fluid to the wound, such as hydrogels and hydrocolloid dressings.^1^

### E (Edge): Assessment of the wound edge

Monitoring the epithelial margin guides the management approach to optimize the necessary conditions. The quality of the wound bed is crucial to facilitating epithelial advancement from the edges.^1^

The edge needs to be assessed to see if it has hyperkeratotic tissue, epibole (rolled edge), or is not advancing, and the edges should be clean and level, as this facilitates epithelial migration.^70^

### R (Repair/Regeneration): Repair/Regeneration

The implementation of advanced therapies for repair should only occur once risk factors are addressed in difficult-to-heal wounds. Some experts limit the use of advanced therapies to wounds that have responded to standard treatment by less than 40%‒50% in four weeks.^1^

Different technologies can be selected based on their suitability for the wound and the patient’s characteristics. These technologies include topical and systemic interventions (Fig. 2).^1^ When epithelial advancement is slow, several alternatives can accelerate the process. In the field of regenerative medicine, based, for example, on tissue engineering, it could be an interesting option in patients with difficult-to-treat ulcers, such as patients with spinal cord injuries.^85^ The findings from the systematic review suggest that cell-based therapies present a promising approach for improving PI outcomes, however, more extensive research is needed on this topic.^86^

The latest technologies for regeneration include mesenchymal and epidermal stem cells, which can be applied to promote tissue regeneration; 3D bio-printing technologies to create biomimetic structures that facilitate healing; genetic therapy and gene editing to modify cellular behaviours; nanotechnology with nanomaterials used as drug carriers or to promote wound healing through controlled release of bioactive substances; and biomimetic scaffolds that develop acellular scaffolds mimicking the extracellular matrix to guide tissue regeneration.^87^

Another therapy that has gained prominence is Platelet-Rich Plasma (PRP). However, it should be noted that it is still in the early stages of use, and possible variations need to be analyzed. A recent systematic review concluded that PRP stands out as a promising and safe therapeutic approach for injuries, but to improve the quality of the evidence, there is a need for new high-quality randomized clinical trials.^88^

### S (Social): Assessment of Social and patient-related factors

It encompasses overall health and well-being, including physical and psychological factors, and the impact on the patient's lifestyle.^1^ Listening to and understanding the patient is key to treatment; involving them in their care and decision-making can improve outcomes and adherence.^89^ The most negative impact on the quality of life of individuals with PIs occurs in the social dimension. PIs affect both patients and their caregivers by limiting their ability to carry out daily activities and making them more dependent on healthcare services and support environments.^90^

The patient should be assessed holistically, including sleep, physical activity, and extrinsic factors such as family and health care facility support. Interdisciplinary actions are essential to alleviate potential problems and risks, particularly related to the proper management of odor and pain, which contribute to low self-esteem, depression, and social stigma.^55^, ^91^

Palliative wound care is a complex discipline that differs significantly from conventional wound treatment, with the primary goal being symptom relief and the provision of empathetic care by healthcare professionals.^92^ Factors that classify a wound as palliative include unmodifiable risk factors or medical conditions such as malnutrition with sarcopenia, inadequate perfusion, multisystem organ failure, immunocompromised states, anasarca, metastatic cancer, and a terminal prognosis that hinders normal healing processes.^69^ A palliative approach can alleviate suffering, enhance quality of life, and reduce healthcare costs by avoiding futile and/or painful procedures and unnecessary expenses.^69^

Education among the patient, family, and healthcare professionals is essential to promote understanding and define the desired care goals.^93^

### PW (Peri-wound): Assessment of the Peri-wound skin

Another current concept has defined the importance of the peri-wound area, even including it at the end of TIMERS as PW (peri-wound).^55^ This area deserves attention, as it may have hyperkeratosis, erythema, maceration, eczema, xerosis, contact dermatitis (both allergic or irritant), or infection.^67^, ^94^ Fig. 2 shows the main measures directed at each condition in this area.

### Surgical approach

Surgical management is usually reserved for recalcitrant wounds or wounds with full-thickness skin loss and exposure of deeper structures such as muscle, fascia or bone (stage 3 and 4 – Table 2).^55^, ^95^, ^96^ Reconstructive surgery usually includes debridement of the wound, followed by filling the wound with new tissue. The surgical approach involves a multidisciplinary team, including the expertise of a plastic surgeon. A systematic review to assess the effects of different types of reconstructive surgery for treating PI, compared with no surgery or alternative reconstructive surgical approaches, included only one randomized clinical trial.^97^ This trial included 20 participants with stage 4 ischial or sacral PI. The study compared two reconstructive techniques: conventional flap surgery and cone of pressure flap surgery, in which a large portion of the flap tip is de-epithelialized and deeply inset to obliterate dead space. The outcomes about complete wound healing, wound dehiscence, PI recurrence and wound infection were collected. The evidence for these outcomes as very low certainty. The authors concluded that there is very little randomized evidence on the role of reconstructive surgery in PI management, although it is considered a priority area. More rigorous and robust research is needed to explore this intervention.

**Table 2:** Specifications and summary of some indications in managing Pressure Injuries (PIs) according to the classification by National Pressure Injury Advisory Panel^7^ and other guidelines and systematic review.^55^, ^95^, ^96^

Summary ‒ PIs classification	Exudate level	Treatment recommendations (remove and treat the cause)	Dressings, products and recommended measures
**Stage 1 PI**			
Non-blanchable erythema, intact skin	Absent	Avoid friction in the area when applying moisturizing creams and during bathing. Focus on pressure relief in the area and daily reassessment.	• Barrier film/cream when required;
			• Five-layer foam dressings with or without silicone adhesive.
**Stage 2 PI**			
Superficial skin loss involving the epidermis/upper dermis.	Intact blister	Keep intact, protecting the area with an appropriate dressing. Depending on the blister's location, size, and characteristics, aspiration with an aseptic technique may be performed to prevent rupture and exposure to infection.	• Foam dressings with or without silicone adhesive; • Collagen, or acrylic dressings
	Broken skin with minimal exudate	Promote treatment with a moist environment but with balance. Pay attention to signs of infection and the color of the exudate.	• Foam dressings with or without silicone adhesive • Hydrocolloid, acrylic dressing, collagen/composite dressing
**Stage 3 PI**			
Full-thickness damage to the subcutaneous tissue, but not reaching the muscle fascia	Low/moderate exudate	Maintain moist treatment. Assess for necrotic tissue requiring debridement and stabilize viable tissues. Manage bacterial balance/biofilm.	• Hydrogel^a^ if autolytic debridement is required. • Antimicrobial gels such as polyhexamethylene biguanide^a^ if local infection. • Absorbent dressings for moderate exudation, such as foam dressings, gelling fibre^b^
	High exsudate	Increased exudate may be indicative of infection. Assess for necrotic tissue requiring debridement and stabilize viable tissues. Manage bacterial balance/biofilm. Protect edges with barrier cream products.	• Superabsorbent dressings, such as polymer foams, silver dressings (e.g., gelling fiber or foam dressings with silver^b^) • Alginate dressings^b^ • Collagen dressings^b^ • Negative pressure wound therapy.
**Stage 4 PI**			
Total loss of skin thickness affecting muscle and/or bone	Low/moderate exudate	Maintain granulation tissue. Assess for necrotic tissue requiring debridement. Manage bacterial balance/biofilm. Be attentive to osteomyelitis (exposed or palpable bone).	• Absorbent dressings for moderate exudation, such as foam dressings, gelling fibre^b^ • Absorbent dressings for infection: foam dressings with silver^b^, gelling fiber dressings with silver^b^, PHMB gel^a^, cadexomer iodine^a^
	High exudate	Increased exudate may indicate of infection ‒ be attentive to osteomyelitis (exposed or palpable bone). Assess for necrotic tissue requiring debridement and stabilize viable tissues. Manage bacterial balance/biofilm. Protect edges with barrier cream products	• High-absorption dressings, such as foams and silver ion dressings^b^ • Negative pressure wound therapy.
**Non-classifiable PI**			
Any wound completely covered by necrotic tissue with no visualization of the wound bed.	Absent	Facilitate debridement (if the wound has the capacity to heal) or protect (keep the necrotic tissue dry if healing is not the goal of care of the wound is ischemic (DIP stages C). Instrumental debridement if there is adequate blood circulation and the patient is not anticoagulated. Combine debridement methods. Pay attention to patient pain.	• Below knee – aim to keep the wound dry until vascular assessment is determined. Apply povidone iodine 10%^a^ to the wound bed. Keep the wound dry until the vascular assessment is determined (exclude ischemic injury); after that, it is possible to perform surgical debridement • Above knee ‒ consider an antimicrobial gel (polyhexamethylene biguanide)^a^ to aid in autolytic debridement • In case of mild bleeding, use calcium and sodium alginate dressing^a^ for hemostasis, or a hemostatic product. In case of severe bleeding seek help from a trained surgeon.
**Deep Tissue PI**			
Purplish, bruised, or blood-filled blister lesion in an area of bony prominence	Absent	Avoid friction in the area when applying moisturizers and during bathing. Focus on relieving pressure in the area and perform daily reassessment.	• Skin protectors • Barrier/film cream • Silicone foam dressings
	Superficial rupture with exudate (may occur days after the onset of the pressure injury)	Maintain moist treatment. Assess the necrotic tissue and proceed with caution when debriding. In cases of deep ischemia, avoid debridement in areas with compromised circulation; If necessary, do so gently and superficially, preserving viable tissue	Depending on the exudate: • Silicone foams for atraumatic coverage, or • Absorbent dressings for moderate exudation, such as gelling fiber and foam dressings.
	Blood blister	Inspect injury for improvement or deterioration and monitor capillary return; Aspiration with an aseptic technique may be performed to prevent rupture and exposure to infection	• Five-layer silicone foam dressing • Absorbent dressing if there is heavy exudate
**Medical Device-Related PI**			
It has the shape of the device and follows the same classification as above, and the same treatment according to depth and location.	Exudate varies according to the stage of involvement and the phase of healing.	Control moisture. Focus on the ease of application and removal of the dressing. Allow assessment of skin condition. Evaluate for infection/biofilm.	• Treatment according to the location and stage of the pressure injury. • Combine wound coverage with protection of the medical device. • Include the use of cushions, coverings, or protective materials that not only prevent additional pressure in the area but also allow for easy and safe access to the device, minimizing the risk of contamination and infection
**PI on mucous membranes**			
Typically caused by medical devices on mucous membranes in areas such as the vaginal, rectal, urethral, and oral cavities.	Absent or exudative	Non-blanchable erythema is not visible on mucous membranes. Check for fluid accumulation. Reposition and adjust medical devices regularly to prevent prolonged pressure on a single point. Remove the device as soon as clinically possible. Assess for fungal/yeast infection.	Mucosa: • Carefully clean the lesion with water or saline and solutions appropriate for mucous membranes, avoiding irritants. • Apply solutions appropriate for the area. • Use topical/systemic antimicrobials as needed. Skin: • Superficial area on the nostril: keep the area protected and consider using gentle white paraffin. • If there is deeper tissue loss and exudate needs to be controlled, consider using a silicone foam dressing. • Necrotic tissue: keep intact and protect with a barrier dressing.

a Interventions that require a secondary dressing, such as: rayon dressing with gauze pads and polyurethane films.

b Interventions that require a secondary dressing, such as: adhesive foam dressing, gauze pads with polyurethane films.

### Indications for dressings and products for pressure injuries according to their classification

It is important to emphasize that the choice of any dressing or product should consider TIMERS, the location and size of the lesion, particularly in advanced therapies. Another consideration is the cost-effectiveness and making choices that are appropriate and consistent with the patient's social condition and treatment goals, with a focused approach whenever relevant to infection/biofilm, as many products are incompatible and should not be used on infected wounds.^1^

Based on guidelines,^7^, ^55^ the most used indications according to the classification of PIs were summarized in Table 2. An algorithm based on the Depth, Infection, and Perfusion (DIP) classification is also useful to clinicians in the initial evaluation, classification, and management of PI.^95^, ^96^

## Prognosis and complications of pressure injuries

**Fig. 3:**
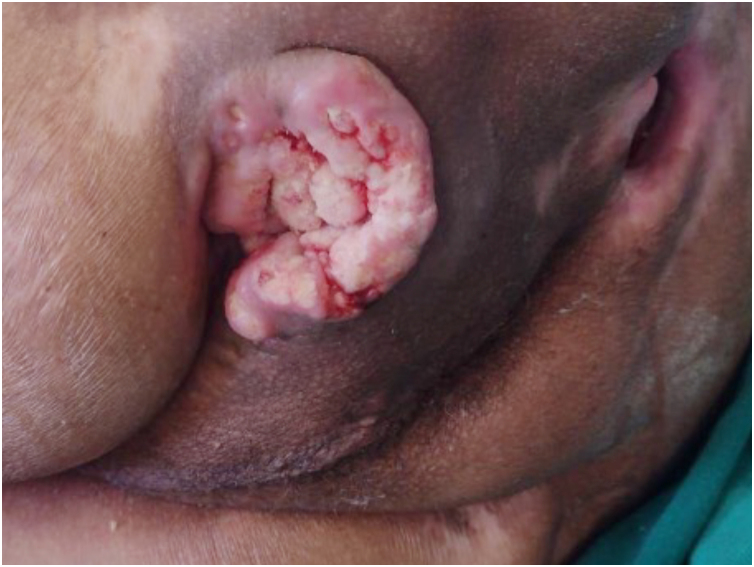
Marjolin's ulcer in a young paraplegic patient with a pressure injury in the ischial location for 15-years.

The occurrence of PIs is generally related to a prolonged period of hospitalization, and patients who develop them have a higher likelihood (22.5%) of not surviving.^39^ Moreover, PIs are associated with various short- and long-term complications, including infection, malignant transformation (Marjolin's ulcer – Fig. 3), fistulas to nearby structures and organs, heterotopic ossification, systemic amyloidosis due to chronic inflammatory state (causing symptoms such as fatigue, weight loss, swelling, cardiac problems, renal dysfunction, neuropathy, and gastrointestinal issues), rhabdomyolysis due to prolonged pressure and ischemia, as well as recurrence of the PIs themselves^98^ and even death.

The most common and severe complication of PIs is infection, which can include cellulitis, abscess, osteomyelitis, bursitis, septic arthritis, necrotizing fasciitis, sepsis, and even death. Often, it is caused by multiple microorganisms and exhibits resistance to various medications and antimicrobials, forming a biofilm, which hinders healing by preventing the migration of keratinocytes.^77^

## Multidisciplinary team

Due to the broad impact, PIs require a multidisciplinary approach. As discussed, PIs are an adverse event that can occur due to various factors. Evidence-based knowledge of these risk factors is essential for all healthcare professionals, as optimal prevention and treatment can only be ensured through close and reliable collaboration.^99^ Communication between the medical team and the nursing team is crucial to ensure awareness of conditions or symptoms relevant to the development of PIs, such as diabetes, hypoalbuminemia, anemia, and hypotension.^99^ In addition to the medical and nursing teams, physical therapists are essential in treating PI by using techniques to improve mobility, reduce pressure on vulnerable areas with special beds and mobilization supports, and assist with self-care.^7^ Dietitians and other multidisciplinary team members are important for holistic care and attention to patients with PIs.^7^ The expertise of a plastic surgeon is needed for reconstructive surgical approaches such as local flaps for recalcitrant PI or those in stages 3 and 4. Dermatologists are essential to performing differential diagnoses and biopsies, recommending appropriate care for the surrounding skin, and indicating proper treatments and specialized dressings.^100^

## Final considerations

This review has expanded knowledge and evidence-based practices for adequately managing patients at risk and those with PIs, detailing current and innovative therapies and conventional ones.

Some key points of this review include: (1) The risk assessment for developing PIs should be structured, including a valid and reliable risk prediction scale, a thorough skin assessment, and a critical interpretation of the results; (2) The prevention bundles (risk assessment, moisture control, patient mobility, nutrition, and support surfaces) should be implemented together to impact patient outcomes better than individual interventions. (3) The first step in the care pathway for PIs is to relieve pressure in affected areas and prevent the development of injuries in new areas. (4) The acronym TIMERS is essential for facilitating efficient ulcer care, focusing on rigorous assessment of the wound bed, and implementing interventions to optimize the healing process. (5) PIs are associated with various short- and long-term complications, including infection, malignant transformation (Marjolin's ulcer), fistulas, rhabdomyolysis, as well as recurrence of the PIs themselves and even death.

Hopefully, it will serve as a decision-support tool for healthcare professionals and a practical guide for qualifications and studies in this critical and highly prevalent area.

## ORCID ID

Bruna Cristina Velozo: 0000-0002-3334-8578

Michelle Venâncio Hong: 0000-0002-4684-2196

Larissa Cassiano Bernardo: 0000-0002-2465-8144

Meire Cristina Novelli e Castro: 0000-0002-0590-4127

## Financial support

None declared.

## Authors’ contributions

Bruna Cristina Velozo: Design and planning of the study; literature survey, drafting and editing of the manuscript; critical review of the literature; critical review of the manuscript; approval of the final version of the manuscript.

Michelle Venâncio Hong: Design and planning of the study; literature survey, drafting and editing of the manuscript critical review of the literature; critical review of the manuscript; approval of the final version of the manuscript.

Larissa Cassiano Bernardo: Design and planning of the study; literature survey, drafting and editing of the manuscript; critical review of the literature; critical review of the manuscript; Approval of the final version of the manuscript.

Meire Cristina Novelli e Castro: Design and planning of the study; literature survey, drafting and editing of the manuscript; critical review of the literature; critical review of the manuscript; approval of the final version of the manuscript.

Luciana Patrícia Fernandes Abbade: Design and planning of the study; literature survey, drafting and editing of the manuscript; critical review of the literature; critical review of the manuscript; approval of the final version of the manuscript.

## Research data availability

Does not apply.

## Conflicts of interest

None declared.
